# Ventilators are not the answer in Africa

**DOI:** 10.4102/phcfm.v12i1.2517

**Published:** 2020-07-27

**Authors:** Farai D. Madzimbamuto

**Affiliations:** 1 Department of Anaesthesia and Critical Care Medicine, Faculty of Medicine, University of Botswana, Gaborone, Botswana

**Keywords:** ventilators, oxygen concentrator, COVID-19, district hospital, Oxygen infrastructure, prone patient, critical care

## Abstract

The treatment of severely ill coronavirus disease 2019 (COVID-19) patients has brought the worldwide shortage of oxygen and ventilator-related resources to public attention. Ventilators are considered as the vital equipment needed to manage these patients, who account for 3% – 5% of patients with Covid-19. Most patients need oxygen and supportive therapy. In Africa, the shortage of oxygen is even more severe and needs equipment that is simpler to use than a ventilator. Different models of generating oxygen locally at hospitals, including at provincial and district levels, are required. In some countries, hospitals have established small oxygen production plants to supply themselves and neighbouring hospitals. Oxygen concentrators have also been explored but require dependable power supply and are influenced by local factors such as ambient temperature and humidity. By attaching a reservoir tank, the effect of short power outages or high demands can be smoothed over. The local and regional energy unleashed in the citizens to respond to the COVID-19 pandemic should now be directed towards developing appropriate infrastructure for oxygen and critical care. This infrastructure is education and technology intensive, requiring investment in these areas.

## Ventilators are not the answer in Africa

For medical staff in low-resourced settings in Africa, the challenge will be first making a diagnosis of coronavirus disease 2019 (COVID-19) and then knowing how to manage it. There may not be time or resources to transfer all seriously ill patients to central hospitals. By the time patients are presenting at peripheral (district and provincial) hospitals, it is likely that central hospitals are overwhelmed with cases.

Of the patients diagnosed with COVID-19, 15% are estimated to have essential critical care in hospital, whilst 3% – 5% of patients require advanced critical care.^1^ Those with advanced respiratory distress who have ended up on ventilators were extremely sick and had a high mortality.^1,2^ The experience of managing critically ill patients with COVID-19 in China and Italy led to media and public speculation on the preparedness of other countries for managing the pandemic by the stock of ventilators each had. Ventilators have become a crucial tool needed to fight the COVID-19 pandemic, despite their cost and scarcity. The shortage of ventilators and intensive care capability in Africa has been a significant limitation in saving critically ill lives for some time. Few provincial or district hospitals in African countries have critical care capability in the public sector.^2^

From the perspective of a district hospital, the preoccupation with ventilators is misplaced. Ventilators alone do not save lives. In addition to the functioning machines and equipment, what is required is the whole package: skilled medical and nursing staff to manage the patients, biomedical staff to manage the necessary equipment, a reliable power supply and oxygen.^3^ Ventilators consume vast quantities of oxygen, up to 60 000 L per day per patient. The mortality of patients on ventilators is high, even with the best care available, particularly in older patients and those with co-morbidities.^4,5^ There are considerable opportunity costs in that attention, and resources are focused on the few who benefit from ventilation, at the expense of a larger number of patients who would do well with supportive care and oxygen.

China mobilised thousands of health workers from across the country and thousands of ventilators, a feat that seemed herculean. In Italy, this was achieved by using a regional critical care network of 74 hospitals and intensive care units.^6^ In China, Europe and North America, the oxygen demand was high but was achieved, even for patients needing high-flow oxygen of 60 L/min. Hospitals have oxygen tanks, plant or cylinder replacement systems, and regular supplies from oxygen manufacturers, although even these became stressed at peak use.^3^

From an African perspective, these interventions are not feasible. In normal times, peripheral hospitals manage perioperative patients who require short-term support or patients who require stabilisation before transferring to a higher level, usually central or tertiary hospitals. Once central hospitals are overwhelmed with COVID-19 patients, peripheral hospitals will have to develop local coping strategies. The single most important intervention in severely ill COVID-19 patients in African countries is sustaining oxygen supplies, currently a scarce resource in most hospitals. The challenge lies in how to improve oxygen supplies for peripheral hospitals so that patients could be managed closer to their communities rather than being transferred to overwhelmed central hospital critical care units. Focusing on oxygen therapy is more feasible and likely to save more lives.

In high-income countries, clinicians observed that COVID-19 patients developed a silent hypoxia with pneumonitis without shortness of breath, and therefore they did not realise the seriousness of their condition. By the time they presented to hospitals with breathing problems, their oxygen levels were very low, and they were already in a critical condition. An early warning system is needed so that staff members in peripheral hospitals in Africa can start identifying patients suspected of having COVID-19-related hypoxia on oxygen in order to help them avoid serious complications. Oxygen therapy can be escalated from low flow (2 L/min – 5 L/min) via nasal canulae or face mask to high flow (10 L/min – 15 L/min) with a non-rebreathing mask and addition of a reservoir bag. Higher flows (40 L/min – 60 L/min) may not be sustainable where oxygen supplies are limited but are increasingly being used to delay or prevent intubation.^7,8^

Pulse oximeters are small devices that are clipped to the finger and are especially useful for early detection of hypoxia and abnormal heart rates. Oximeter technology can be made available for every district hospital in Africa, an initiative already in progress through donations made through the Lifebox scheme, facilitated by a global tender issued by the World Federation of Societies of Anaesthesiologists (WFSA) (https://www.lifebox.org/purchase-oximeter). Lifebox pulse oximeters, which are equipped with long-lasting batteries and able to survive severe voltage fluctuations when plugged into electric mains, are relatively inexpensive and suitable for use in under-resourced environments. The Lifebox website houses a decision and triage tool to help clinicians during the COVID-19 pandemic, using oxygen saturation measurements from a pulse oximeter to determine when oxygen support is needed (https://www.lifebox.org/covid-19-decision-tool/).

Another intervention useful in low-resource settings is ‘proning’ or patient positioning manoeuvres (having patients lie on their stomach and sides) which open up the lower and posterior lungs most affected in COVID-19 pneumonia. Studies have shown that oxygenation and positioning improved patients’ breathing and prevented progression of the disease in many cases.^1^

The poor oxygen infrastructure in many African countries needs urgent attention. Few countries manufacture oxygen and rely on importing it. In southern Africa, this would be mainly from South Africa. Oxygen is usually distributed to peripheral hospitals in oxygen cylinders, which is an expensive route. Oxygen concentrators rely on electricity, whose supply is often erratic.^9^ Manufacture of essentials like oxygen from scratch is unlikely to be sufficiently established in time to respond to this epidemic, but the conversation around local manufacture has been escalated. There are several examples of small-scale local production of oxygen supplies for hospitals that can be used as models. Assist International has partnered with governments in several African countries to develop access to oxygen (https://assistinternational.org/global-health/access-to-oxygen/). In January 2020, the Kilimanjaro Christian Medical Centre in Tanzania opened up an oxygen plant to supply itself and distribute some for local hospitals nearby. In Kenya, a similar approach has been used by Hewa Tele (https://hewatele.org/) since 2014 to build an oxygen plant next to a regional hospital and then supply to local hospitals nearby. Similar developments have been seen in Ethiopia and Rwanda.^5,10^

Oxygen concentrators come in portable bedside machines and hospital-sized oxygen plants. They are simple to use but need careful assessment for local suitability.^11,12^ Flow rates are up to 5 L/min – 10 L/min, and the oxygen concentration achieved may vary depending on environmental temperatures, humidity and power fluctuations. Concentrators have been used with reservoir tanks to maintain oxygen supply during short power outages and high demands.^13^

The focus on critical care equipment and its maintenance should also raise discussions about strengthening and extending local qualifications in Biomedical Engineering to enhance the skilled workforce for this field.

In conclusion, COVID-19 has highlighted the deficiencies in critical care management and infrastructure, particularly in provincial and district hospitals. Financial investment in procuring reliable oxygen supplies, oxygen concentrators and oximeters would be far more viable than manufacturing ventilators. Decentralising oxygen production to regional hospitals could lay the basis for critical care infrastructure. This investment would benefit other services for surgery, respiratory illnesses and critical care medicine after the COVID-19 pandemic is gone.
